# Whirl Sign and Midgut Volvulus: An Unusual Cause of an Acute Abdomen in an Adult Patient

**DOI:** 10.1155/2019/2356702

**Published:** 2019-05-06

**Authors:** Eoghan P. Burke, Munir Saeed, Maham Mahmood, Cathal Hayes, Mohamed Salama, Ibrahim Ahmed

**Affiliations:** Department of Surgery, Our Lady of Lourdes Hospital, Drogheda, Ireland

## Abstract

We report the case of a 50-year-old lady who presented to the emergency department complaining of a two-day history of colicky right upper quadrant (RUQ) pain, which radiated through to her back, associated with nausea, anorexia, and two episodes of vomiting that day. She was found to be tender in the RUQ. Her blood tests were notable for an elevated white cell count. Initial impression was of acute cholecystitis. Ultrasound of her abdomen did not identify any features of acute cholecystitis; however, a large volume of free fluid was identified within the abdomen. CT of the abdomen/pelvis was obtained which identified dilated loops of small bowel, interloop ascites, and a whirl sign highly suggestive of midgut volvulus. During laparoscopy, the midgut volvulus was found to have resolved. No cause for the volvulus could be identified, and the patient was discharged home well on postoperative day two.

## 1. Introduction

The term volvulus is derived from the Latin word *volvere* which means to roll [1]. Midgut volvulus occurs when the small bowel twists greater than 180° around its mesentery. This twisting results in compression of the bowel lumen and the vasculature within the bowel wall, which if uncorrected will progress to ischaemia and infarction of the involved segment of small bowel [2]. The twisting of the small bowel around its mesentery often results in a classic *whirl sign* on CT [3]. Midgut volvulus is rare in the adult population but remains an important cause of small-bowel obstruction and one which requires early recognition and prompt intervention.

## 2. Case Report

The patient was a 50-year-old lady who presented to the emergency department with a two-day history of colicky right upper quadrant (RUQ) pain which radiated through to her back. This pain was associated with anorexia, nausea, and two episodes of vomiting that day. The patient reported having a normal bowel motion the previous day and reported passing flatus that day. She denied any pale stool, diarrhoea, or dark urine. She had no significant past medical or surgical history and was on no regular medication.

On physical examination, her abdomen was slightly distended, soft, and tender in the RUQ with reduced bowel sounds. She was Murphy's sign negative. Her vital signs were all within normal limits.

Her blood tests (Table 1) were notable for an elevated white cell count of 18.2 × 10^9^/l.

**Figure 1:**
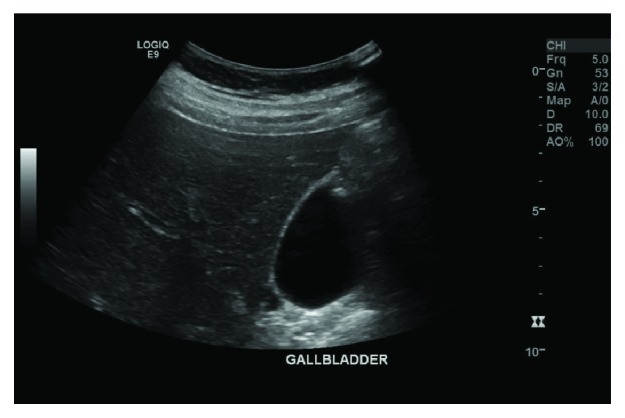
US of the abdomen. Gallbladder with no sonographic evidence of cholecystitis or cholelithiasis.

**Figure 2:**
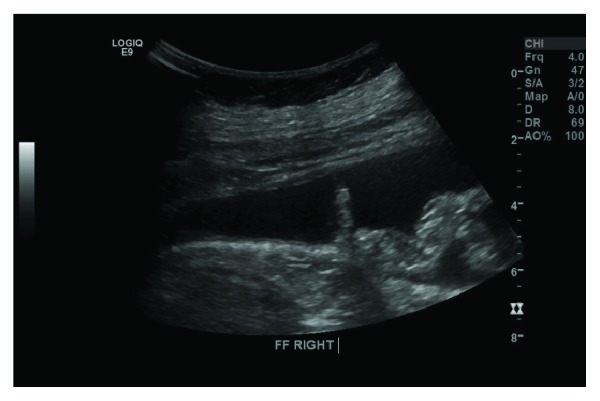
US of the abdomen. Free fluid in the right paracolic gutter.

Initial impression was that of acute cholecystitis, and an ultrasound scan revealed a gallbladder with no evidence of cholelithiasis or cholecystitis (Figure 1). The scan, however, did reveal a large volume of intra-abdominal free fluid (Figure 2).

**Figure 3:**
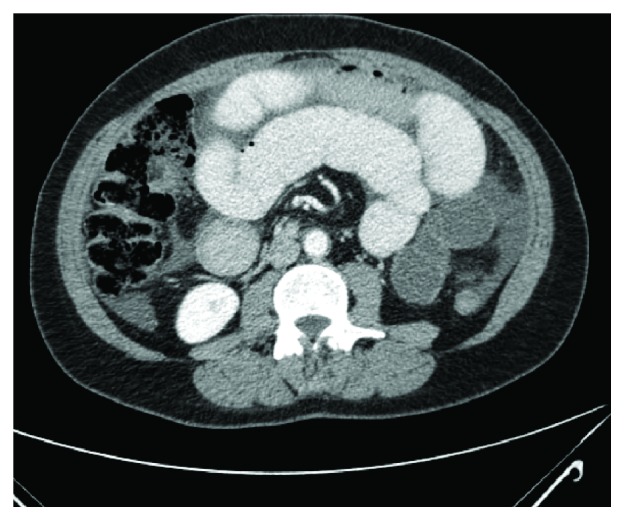
CT of the abdomen. Distended loops of small intestine in the right and left upper and middle quadrants, consistent with small-bowel obstruction. There is an associated moderate volume of interloop ascites within the small-bowel mesentery.

**Figure 4:**
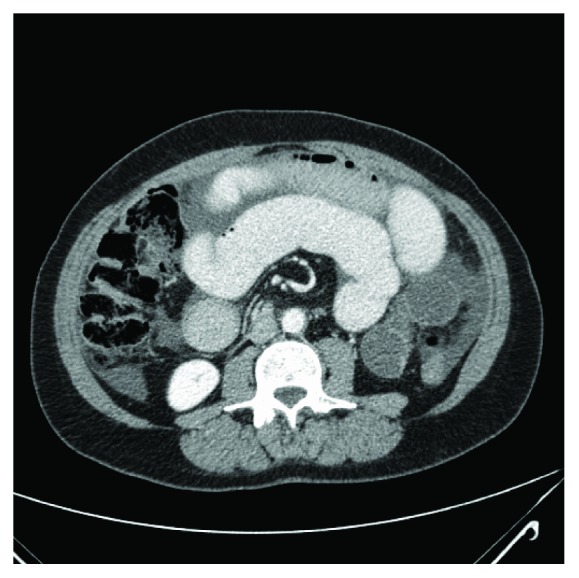
CT of the abdomen. Whirl sign at the base of the mesentery around the superior mesenteric artery consistent with midgut volvulus.

In light of this finding, we obtained a computed tomography (CT) scan. This scan identified multiple dilated loops of small bowel, consistent with small bowel obstruction (Figure 3), and twisting of the small-bowel mesentery around the axis of the superior mesenteric artery (SMA) in a classic *whirl sign* consistent with midgut volvulus (Figure 4).

In light of these findings, it was decided to proceed to laparoscopy. During laparoscopy, there was a large volume of ascitic fluid throughout the abdomen. The distal small bowel was collapsed with the proximal portion distended. No volvulus was encountered. There were scattered haemorrhagic areas identified along the small bowel. Evaluation for a cause of the midgut volvulus, including a particularly long mesentery, did not yield any results.

The patient was gradually returned to a normal diet and was discharged home on postoperative day two with no long-term complications at 6-month review at the clinic.

## 3. Discussion

Midgut volvulus occurs when the small bowel rotates greater than 180° around its mesenteric vascular pedicle. Midgut volvulus is considered a surgical emergency which often warrants surgical intervention to prevent the progression to small-bowel infarction and the associated morbidity and indeed mortality.

Broadly speaking, midgut volvulus can be classified into primary or secondary. Primary midgut volvulus is typically seen in young children and no predisposing cause is identified during laparotomy/laparoscopy. Secondary midgut volvulus is usually seen in elderly patients (sixth to eight decade of life) and is associated with an underlying congenital or acquired cause [4].

The clinical presentation of primary midgut volvulus is usually nonspecific, and reports exist in the literature of midgut volvulus mimicking other intra-abdominal pathologies including acute appendicitis [5]. An acute onset of signs or symptoms suggestive of small-bowel obstruction in any patient with no previous surgeries and no hernia identified on physical examination should raise suspicion for this entity.

Initial evaluation of any patient with a suspected primary midgut volvulus should include plain film abdominal radiographs which may demonstrate nonspecific signs of small-bowel obstruction including dilated small-bowel loops and air fluid levels and occasionally may identify signs of intestinal ischaemia including pneumatosis intestinalis and thumbprinting [6].

During ultrasound, it is often possible to identify the rotation of the small-bowel loops and superior mesenteric vein (SMV) around the superior mesenteric artery—the so-called “whirlpool sign” which is highly suggestive of midgut volvulus. Some reports would indicate that US can detect midgut volvulus with 92% sensitivity and 100% specificity [7].

Currently, CT scanning is considered the imaging modality of choice as it allows for identification of the pathognomonic *whirl sign* of midgut volvulus. The *whirl sign* denotes the pattern of the mesentery and SMV rotated clockwise around the SMA [8].

Clinically suspected and certainly radiologically confirmed midgut volvulus mandates surgical intervention to rule out gangrenous bowel. There remains much debate as to the correct management of primary midgut volvulus in adults. Devolution of the volvulus and resection of any nonviable bowel should be performed in all cases; however, some advocate for fixation of the small bowel in an attempt to prevent future recurrence [9].

## Figures and Tables

**Table 1:** Blood test results in the emergency department.

Parameter	Units	Reference range
White cell count	18.2 × 10^9^/l	4.0-11.0
C-Reactive protein	2.5 mg/l	0.0-5.0
Total bilirubin	10 *μ*mol/l	3.4-20.5
Alkaline phosphatase	54 IU/l	40-150
Gamma GT	28 IU/l	9-36
ALT	19 IU/l	<55
Amylase	32 IU/l	25-125
Creatinine	57 *μ*mol/l	50-98
Urea	4.0 mmol/l	2.5-6.7
